# High-throughput screening for tick-borne pathogens in Ixodid ticks collected through crowdsourcing in northern Sweden

**DOI:** 10.1186/s13028-026-00854-9

**Published:** 2026-02-18

**Authors:** Giulio Grandi, Seungeun Han, Karin Ullman, Ann Albihn, Sara Moutailler, Clémence Galon, Linnea Öborn, Phimphanit Choklikitumnuey, Ann Högberg, Galina Ganchenko, Anton de Jong, Anna Omazic

**Affiliations:** 1 Department of Animal Biosciences, Swedish University of Agricultural Sciences (SLU), Uppsala, Sweden; 2 Department of Epidemiology and Disease Control, Swedish Veterinary Agency (SVA), Uppsala, Sweden; 3 Department of Microbiology, Swedish Veterinary Agency (SVA), Uppsala, Sweden; 4 Laboratoire de Santé Animale, ANSES, INRAE, Ecole Nationale Vétérinaire d’Alfort, UMR BIPAR, Maisons-Alfort, France; 5 Department of Chemistry, Environment and Feed Hygiene, Swedish Veterinary Agency (SVA), Uppsala, Sweden

**Keywords:** *Anaplasma* spp., *Borrelia* spp., Citizen science, Ixodes persulcatus, Ixodes ricinus, *Rickettsia* spp., Ticks, Tick-borne pathogens

## Abstract

**Background:**

Ticks are expanding in the northern hemisphere. Along with them, tick-borne pathogens can be introduced into new geographical areas and cause infection and disease in animals and humans. Monitoring the expansion of tick populations is challenging and in large areas such as northern Sweden it can be beneficial to take advantage of citizen science. Therefore, people living in northern Sweden were asked to submit ticks collected from their pets or from themselves during the tick seasons of 2018 (north of river Dalälven; *n* = 1087) and 2019 (from the four northernmost Swedish provinces; *n* = 514). Ticks were identified at the species level and further analysed with a microfluidic technique to detect carried tick-borne pathogens. Forty-eight PCR assays targeting an array of tick-borne bacteria, viruses and protozoa were performed per sample in the assay.

**Results:**

The most frequently detected pathogens were *Rickettsia helvetica* (15.6% in 2018 and 3.5% in 2019) followed by *Borrelia garinii* (5.9% in 2018 and 11.5% in 2019) and *Borrelia afzelii* (5.7% in 2018 and 1.2% in 2019).

**Conclusions:**

This study provides data on tick-borne pathogens harbored by feeding ticks collected from a rather poorly investigated geographical area using a One Health perspective. Microfluidic techniques are confirmed to be an effective tool to screen large amounts of samples and to also find pathogens occurring at lower rates. This approach best supports the design of updated risk-maps and to find areas that deserve targeted tick sampling to obtain a more accurate risk assessment and achieve effective disease prevention.

**Supplementary Information:**

The online version contains supplementary material available at 10.1186/s13028-026-00854-9.

## Background

Ixodid ticks (Acari: Ixodidae) are known as the primary vectors for several pathogens of medical and veterinary importance worldwide [1]. In the Palearctic region, *Ixodes ricinus* and *Ixodes persulcatus* play an important role in human and veterinary public health as vectors of several tick-borne pathogens (TBPs) [2–4]. While *I. ricinus* has been considered as an established tick species in southern and central Sweden during the last decades (even if expanding northwards), *I. persulcatus*, probably introduced by birds, has become established in northern Sweden only recently [5]. Moreover, this species has enlarged its distributional area and population density during the last five years [6]. Based on results from our recent citizen science studies, beside the two abovementioned generalist species, people in northern Sweden also encountered a nidicolous species, *Ixodes trianguliceps* [6], although to a lesser extent, and only sporadically encountered *Hyalomma* spp [7].

Being generalist tick species, both *I. ricinus* and *I. persulcatus* allow the circulation of a rather wide array of TBPs, reflecting the broad host range of these tick species (mammals, birds and reptiles) [8]. The TBPs transmitted by these species and considered most relevant for human health are the spirochetes belonging to genus *Borrelia* (especially the three species associated to Lyme disease i.e. *Borrelia burgdorferi* s.s., *Borrelia afzelii* and *Borrelia garinii*, and the relapsing fever spirochete, i.e. *Borrelia miyamotoi*) and the virus causing tick-borne encephalitis (TBEV) [9–11]. Other bacterial TBPs that *I. ricinus* and *I. persulcatus* can carry are several rickettsiae, *Anaplasma phagocytophilum*, *Neoehrlichia mikurensis*, *Francisella tularensis*, *Bartonella* spp. along with the protozoa of the genus *Babesia* [2, 8]. Besides TBEV, other viral TBPs of significant medical concern potentially transmitted by *I. ricinus* and *I. persulcatus* are Uukuniemi virus (UUKV) [12, 13], Louping ill virus (LIV) and Eyach virus (EYAV) [14, 15]. Most TBPs carried by these two species can be considered zoonotic, but traditionally only *A. phagocytophilum* has been considered a primary animal pathogen [16]. Even *N. mikurensis* and TBEV have been associated with clinical cases in animals, especially in dogs [17, 18] and horses [19, 20]. *I. trianguliceps*, despite feeding mainly on rodents and small insectivores and only rarely on humans, can contribute to the circulation of several TBPs, including *Babesia microti*, *A. phagocytophilum*, *B. garinii*, *B. afzelii*, *F. tularensis* and TBEV [8].

The effects of climate change on tick abundance and geographical distribution in northern Europe have been studied on many occasions [6, 21–24], and some studies have focused on the impact of climate change on the incidence of the major tick-borne diseases (TBDs), especially Lyme disease and TBEV [25–28]. Regarding Lyme disease, it seems that climate change has led to an increased incidence of the disease in Europe [27, 29] and in North America [30, 31], while regarding TBEV there are contradictory opinions since different modeling approaches has provided different results [32–34]. The impact of climate change on other TBDs has not been deeply studied, even if there are some indications that even the incidence of human babesiosis might increase due to climate change [35].

The increasingly frequent use of molecular techniques for the detection of TBPs has led to the availability of abundant literature reporting their occurrence in many tick species, including *I. ricinus*, where both ticks collected from the environment and/or from the host have been examined. The majority of these studies have focused on one or a few TBPs, representing an excellent source of information for a given TBP, but at the same time making it difficult to obtain a complete picture of which TBPs a given tick population can harbor at the same time. This was also the case of Sweden, where the occurrence of the major TBPs in ticks has been extensively studied [36–44]. In recent studies, this limitation was overcome by using high-throughput microfluidic techniques, that allow to identify the presence of an array of tick-borne bacteria, viruses and protozoa, both those that are expected to be present but also those that could be introduced from bordering regions or simply considered as emerging [45–51]. In the case of relatively new or poorly investigated tick populations, this tool is particularly advantageous since it can provide a rapid screening of the occurrence of TBPs in the study area. To the authors’ knowledge, this is the first study on TBPs detection in host-collected ticks in northern Sweden performed using this approach. Besides providing information on the occurrence of 48 different TBPs, this study provides also information on their geographical distribution within the study area, and on their association with three major host species considered, i.e. dogs, cats, and humans.

## Methods

### Tick collection

**Fig. 1:**
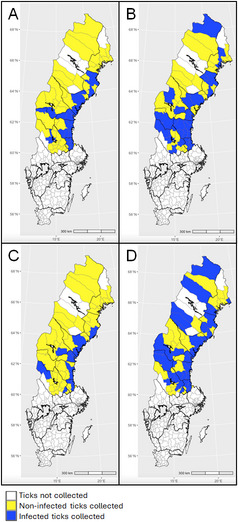
Geographic distribution of tick-borne pathogens. **A** *Borrelia garinii*, **B** *Borrelia afzelii*, **C** *Anaplasma phagocytophilum*, **D** *Rickettsia helvetica—*in *Ixodes ricinus* collected from 11 northern provinces in Sweden in 2018

**Fig. 2:**
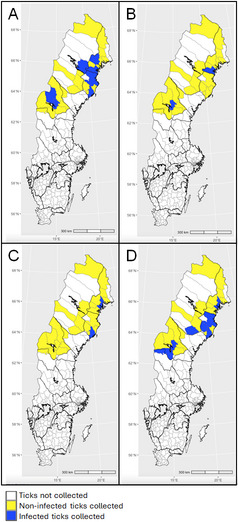
Geographic distribution of tick-borne pathogens. **A** *Borrelia garinii*, **B** *Borrelia afzelii*, **C** *Anaplasma phagocytophilum*, **D** *Rickettsia helvetica—*in *Ixodes ricinus* collected from 4 northern provinces in Sweden in 2019

**Fig. 3:**
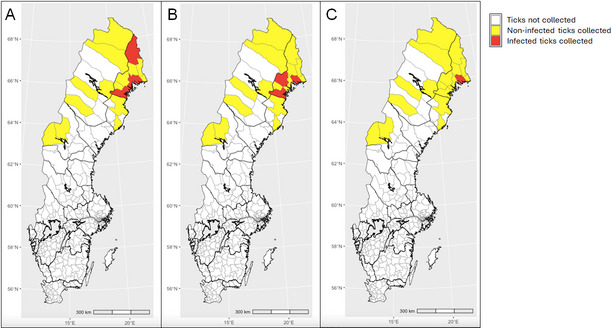
Geographic distribution of tick-borne pathogens. **A** *Borrelia garinii*, **B** *Borrelia afzelii*, **C** *Rickettsia helvetica—*in *Ixodes persulcatus* collected from 4 northern provinces in Sweden in 2019

Ticks were collected with help of citizen science during June to October in two consecutive years (2018 and 2019) in northern Sweden [6]. In 2018, the collection covered municipalities in the provinces located north of the river Dalälven, one of the geographical borders traditionally dividing the northern region of Sweden (Norrland) from its central and southern regions (Fig. 1). In 2019, the collection was focused on municipalities in Sweden’s northernmost provinces (i.e. Lappland, Norrbotten, Västerbotten and Jämtland; Figs. 2 and 3). The chosen study area is considered the most interesting for monitoring the northern expansion of *I. ricinus* and the population growth of *I. persulcatus*.

The Swedish Veterinary Agency (SVA), Uppsala, Sweden, issued a press release in early June 2018 and 2019 to inform the public in the study area about the study. Information about the collection was also disseminated with the help of national media channels and social media. Citizens living in or visiting the study area could send ticks found either on themselves or on domestic and wild animals between June and October. Tick samples were sent by post to SVA, where all the ticks were registered with information about the municipalities from where they had been submitted and the host species from which they were collected. Individual ticks were stored at −80 °C pending molecular analysis of TBPs. Samples with incomplete information (municipality of origin, host species) or severely damaged specimens were not processed further. In 2018, a subset of 1,087 ticks out of a total of 3,131 ticks were screened for TBPs. The corresponding number for 2019 was a subset of 514 ticks out of a total of 558 submitted ticks.

Ticks were morphologically identified using a stereomicroscope (Leica MZ16, Leica Microsystems, Stockholm, Sweden) with up to x200 magnification. Morphological taxonomic keys and illustrations were used for species identification [8, 52]. Criteria for the morphological stage and species identification of the collected ticks, including the molecular differentiation between *I. ricinus* and *I. persulcatus* have been previously described in Omazic et al. [6].

### Extraction of nucleic acids and cDNA synthesis

The ticks were individually washed for 1 min in 70% ethanol solution followed by MilliQ water in preparation for further molecular analyses. Each individual tick was placed in a 2-mL screw-cap micro tube with 450 µL lysis buffer solution, which comprised 441 µL Buffer RLT (Qiagen, Hilden, Germany) and 9 µL 2 M Dithiothreitol (DTT) (Sigma-Aldrich, St Louis, MO, USA), plus a 5 mm stainless steel bead (Qiagen, Hilden, Germany). The ticks were then homogenized for two cycles of one min each either in a Tissue Lyser (Qiagen, Hilden, Germany) at a frequency of 30 Hz or with FastPrep-24 (MP Biomedicals, Santa Ana, California, USA) at 6.5 m/s. The tubes were centrifuged for 3 min at 20,000 × g and 90 µL of the supernatants were incubated with 10 µL of Proteinase K (P4850; Sigma-Aldrich, St Louis, MO, USA) for at least 10 min in a 96-well plate. Positive and negative controls were included on each plate. The positive control consisted of 5 µL of *B. afzelii* (strain Lu81, 10^8^ cells/ml) and 5 µL inactivated TBEV (strain K23, Encepur^®^, Chiron Vaccines, Marburg, Germany) in 90 µL lysis buffer solution and the lysis buffer solution alone was used as a negative control. The extraction of total nucleic acids (NA) was performed in the Magnatrix 8000+ extraction robot (Magnetic Biosolutions, Stockholm, Sweden) with one of two commercial extraction kits–either the Bullet Stool Kit 1.32.104 (DiaSorin, Saluggia, Italy) or the Vet NA kit 1.001 (Bioservices, Stockholm, Sweden)–with an elution volume of 60 µL.

The eluted NA was reverse-transcribed to cDNA using Illustra™ Ready-to-Go RT-PCR Beads kit (GE Healthcare, Amersham Place, UK). Twenty µL NA and 10 µL pd(N)6 random hexamer primers (0.25 µg/µL) were incubated for 5 min at 97 °C and then mixed with one RT-PCR bead dissolved in 20 µL Rnase-free water. The mixture was incubated for 30 min at 42 °C, followed by 5 min at 97 °C, producing 50 µL cDNA. A combination of equal volumes of total NA and cDNA (NA-cDNA) was used for the pre-amplification and microfluidic real-time PCR.

### Pre-amplification and high-throughput microfluidic real-time PCR

The ticks were screened for microorganisms and tick species using chips with 48.48 dynamics arrays in a Bio-Mark™ real-time PCR system (Standard BioTools, San Francisco, California, USA) enabling 48 samples to be analyzed in 48 PCRs simultaneously on each chip. Development and validation of the high-throughput microfluidic real-time PCRs have been previously described [50, 51, 53]. Detailed information about primers and probes used for pre-amplification and the subsequent high-throughput microfluidic real-time PCR is shown in Additional file 1 (new designs developed for this study were firstly tested with positive controls–culture or plasmid–through classical real-time PCR as described in [50, 51, 53]). The PCR reactions included in the chip comprise detection of 18 species and 5 genera of bacteria (*Borrelia* spp., *B. burgdorferi* s.s., *B. garinii*,* B. afzelii*,* B. miyamotoi*,* Borrelia valaisiana*,* Borrelia lusitaniae*,* Borrelia spielmanii*,* Borrelia bissettii*,* Borrelia duttoni*,* Borrelia recurrentis*,* Borrelia bavariensis*,* Anaplasma* spp., *Anaplasma phagocytophilum*,* Ehrlichia* spp., *Ehrlichia chaffeensis*,* Neoehrlichia mikurensis*,* Rickettsia* spp., *Rickettsia slovaca*,* Rickettsia helvetica*,* F. tularensis*,* Coxiella burnetii*,* Bartonella* spp.), 7 species and 2 genera of parasites (*B. microti*,* Babesia venatorum*,* Babesia divergens*,* Babesia major*,* Babesia vogeli*,* Babesia vulpes*,* Toxoplasma gondii*,* Hepatozoon* spp., *Theileria* spp.*)*, 10 virus species (TBEV European subtype, LIV, Powassan virus, Omsk hemorrhagic fever virus, EYAV, Crimean-Congo hemorrhagic fever virus, Hazara virus, UUKV, Schmallenberg virus (SBV), African swine fever virus) and 2 tick species (*I. ricinus*,* I. persulcatus)*. One reaction targeting the conserved region of the 16 S rRNA gene in ticks served as an extraction control. DNA from *Escherichia coli* (EDL933 strain) was added to each chip for detection of the *eae* gene to detect potential inhibition. Each chip also contained a negative water control.

The pre-amplification was performed on all samples to enrich DNA from the microorganisms compared to tick DNA. All the primers (except those targeting tick species DNA (*I. ricinus*,* I. persulcatus)* and *E. coli*) were pooled to a final concentration of 200 nM each and 1.25 µL of this mixture was used together with 1 µL PreAmp Master Mix (Standard BioTools, San Francisco, California, USA), 1.25 µL NA-cDNA and 1.5 µL MilliQ-water in a final volume of 5 µL. The thermocycling program consisted of one cycle at 95 °C for 2 min, 14 cycles at 95 °C for 15 s and 4 min at 60 °C. At the end of the cycling program the reactions were diluted 1:5 in Milli-Q water. The pre-amplified DNA was stored at −20 °C until use.

High-throughput microfluidic real-time PCR was performed using TaqMan Gene Expression Master Mix (Applied Biosystems, Foster City, CA, USA) according to the manufacturer´s instructions with the cycling conditions: 2 min at 50 °C and 10 min at 95 °C followed by 40 cycles of a two-step amplification for 15 s at 95 °C and 1 min at 60 °C. The TaqMan probes were labeled with 6-carboxyfluorescein (6-FAM) and Black Hole Quencher (BHQ1). Data were acquired on the BioMark™ Real-Time PCR System and analyzed using the Fluidigm Real-Time PCR Analysis software to obtain crossing point (CP) values. The cut-off crossing point was set to ≤ 30.

### Confirmation of BioMark™ real-time PCR results by sequencing

In order to evaluate, refine and validate the results obtained with the BioMark™ system, conventional PCRs were performed on a subset of representative positive samples as described in [49]. This representative subset included at least 20% of all positives to each pathogen. For validation of the microfluidic real-time PCR assays, only samples showing low Cq values (< 30) were subjected to conventional PCR assays. Different primers were used for each microorganism compared to those in the microfluidic real-time PCR, following published thermal protocols [49]; more details are provided in Additional file 2. For sequencing, the generated PCR products were purified using ExoSAP-IT™ *Express* PCR Product Cleanup (Applied Biosystems, Foster City, CA, USA) and sent for Sanger sequencing at Macrogen (Macrogen Europe B.V., Amsterdam, The Netherlands). The generated chromatograms were edited and assembled using the BioEdit Software v.7.2. (Hall T.A., 1999). Consensus sequences were analyzed using Basic Local Alignment Search Tool (BLAST) at the website of the National Center for Biotechnology Information (NCBI) (https://ncbi.nlm.nih.gov/blast), compared to reference sequences listed in the GenBank sequence database (NCBI). For each pathogen, only one sequence (the most representative one) appears and is presented in Additional file 3. Moreover, all sequences obtained were aligned, and if they were identical, only the longest one was submitted. If we obtained different sequences after alignment, even those were submitted. GenBank accession numbers of the obtained sequences are available in the same file.

Sequencing results were interpreted as follows: (i) samples that could successfully be sequenced after the PCR for a given species allowed to confirm and extrapolate the results obtained by microfluidic PCR for that same species; (ii) if no sequencing was obtained, all the samples for that species were declared negative; and (iii) if, during sequencing, the quality of the sequence obtained did not allow confirmation at the species level of a given TBP, the samples in question were considered positive but only at the genus level.

### Detection of subtypes of TBE-virus

The PCR reaction included in the microfluidic PCR system was only targeting the European subtype of TBEV. Therefore, all tick samples identified as *I. persulcatus* (*n* = 18 in 2018 and *n* = 275 in 2019) were also screened for all three subtypes of TBEV using two additional real-time PCR assays [54, 55], since this tick species is a potential vector for the Far Eastern and Siberian subtypes of the virus.

### Data management and statistical analyses

Direct estimates of percentage were used for categorical variables, i.e. prevalence of TBPs positive ticks or proportion of collected ticks from specific host species. Infection prevalences were accompanied by 95% confidence intervals (CI) in brackets. We used Chi-square tests to evaluate differences of prevalence among life stages and sex of ticks and results were considered significant at *p* ≤ 0.05.

## Results

### Pathogen occurrence

In 2018, a total of 1,087 ticks, consisting of 1,059 *I. ricinus*, 18 *I. persulcatus* and 10 *I. trianguliceps*, were collected from 64 municipalities in 11 provinces in Sweden. Positive PCR reactions (*n* = 443) were obtained from 368 (33.9%, CI: 31.1–36.7) ticks infected with one up to four different pathogens per tick of the following TBPs: *B. garinii*, *B. afzelii*, *B. valaisiana*, *B. miyamotoi*,* Anaplasma* spp., *A. phagocytophilum*, *N. mikurensis*, *Rickettsia* spp., *R. helvetica*, *F. tularensis*, *B. microti*, *B. venatorum*, *B. divergens* or UUKV. Infected ticks were collected from 80% (*n* = 52) of the municipalities in 11 provinces in the study area.

Specimens of *Ixodes ricinus*, the dominant tick species, were collected from 51 municipalities and consisted of 34 nymphs, 918 females and 107 males; 34.0% (31.2–36.9, *n* = 360) of the specimens harbored nucleic acids of one up to two different TBPs. Infection rates among nymphs (38.2%, 23.9–55.0, *n* = 13), females (33.1%, 30.1–36.2, *n* = 304) and males (40.2%, 31.4–49.7, *n* = 43) of *I. ricinus* did not differ (*χ*^*2*^ = 2.4172, *df* = 2, *P* = 0.2986). The pathogen detected most frequently was *R. helvetica*, followed by *B. garinii*, *B. afzelii* and *Rickettsia* spp. Further, *B. garinii*, *N. mikurensis*, *R. helvetica*, and *Rickettsia* spp. were detected from *I. persulcatus*. *Ixodes trianguliceps* ticks were infected with *A. phagocytophilum*, and *Rickettsia* spp. Results are summarized in detail in Table 1. The geographical distribution of the two most common *Borrelia* species, as well as the geographical distribution of *A. phagocytophilum* and *R. helvetica* infections in *I. ricinus* collected in 2018 is presented in Fig. 1, and details of infected ticks identified in each province are provided in Additional file 4.

**Table 1:** The occurrence of tick-borne pathogens in Ixodes spp. Ticks collected in 2018

2018 Sample Pathogen	Sample		I. ricinus								I. persulcatus								I. trianguliceps					
			Nymph		Female		Male		Total		Nymph		Female		Male		Total		Nymph		Female		Total	
	(*n* = 1087)	%	(*n* = 34)	%	(*n* = 918)	%	(*n* = 107)	%	(*n* = 1059)	%	(*n* = 1)	%	(*n* = 13)	%	(*n* = 4)	%	(*n* = 18)	%	(*n* = 1)	%	(*n* = 9)	%	(*n* = 10)	%
*Borrelia garinii*	64	5.9%	2	5.9%	48	5.2%	13	12.1%	63	5.9%			1	7.7%			1	5.6%						
*Borrelia afzelii*	62	5.7%	5	14.7%	52	5.7%	5	4.7%	62	5.9%														
*Borrelia valaisiana*	15	1.4%			12	1.3%	3	2.8%	15	1.4%														
*Borrelia miyamotoi*	4	0.4%			4	0.4%			4	0.4%														
*Anaplasma phagocytophillum*	21	1.9%			16	1.7%	3	2.8%	19	1.8%											2	22.2%	2	20.0%
Other Anaplasma spp.^*^	1	0.1%			1	0.1%			1	0.1%														
*Neoehrlichia mikurensis*	26	2.4%			24	2.6%	1	0.9%	25	2.4%			1	7.7%			1	5.6%						
*Rickettsia helvetica*	170	15.6%	6	17.6%	141	15.4%	20	18.7%	167	15.8%			3	23.1%			3	16.7%						
Other Rickettsia spp.^**^	38	3.5%			31	3.4%	4	3.7%	35	3.3%					1	25.0%	1	5.6%			2	22.2%	2	20.0%
*Francisella tularensis*	1	0.1%			1	0.1%			1	0.1%														
*Babesia microti*	4	0.4%			4	0.4%			4	0.4%														
*Babesia venatorum*	18	1.7%	2	5.9%	15	1.6%	1	0.9%	18	1.7%														
*Babesia divergens*	3	0.3%			3	0.3%			3	0.3%														
*UUKV*	16	1.5%			13	1.4%	3	2.8%	16	1.5%														
Total infections	443	40.8%	15	44.1%	365	39.8%	53	49.5%	433	40.9%			5	38.5%	1	25.0%	6	33.3%			4	44.4%	4	40.0%

* Excluding samples positive for *Anaplasma phagocytophilum*

**Excluding samples positive for Rickettsia helvetica

In 2019, a total of 514 ticks, consisting of 239 *I. ricinus* and 275 *I. persulcatus*, were collected from 32 municipalities in the four northernmost Swedish provinces. *Ixodes ricinus* (nymphs, *n* = 2, females, *n* = 221 and males, *n* = 16) showed an infection rate of 17.2% (12.9–22.4, *n* = 41) with one to two different pathogens per tick of the following TBPs: *B. garinii*, *B. afzelii*, *B. miyamotoi*, *A. phagocytophilum*, *N. mikurensis*, *R. helvetica*, *Rickettsia* spp., and *B. venatorum*. Infection rate of male *I. ricinus* (*n* = 7) was significantly higher than that of females (*n* = 33; *χ*^*2*^ = 8.832, *df* = 1, *P* = 0.003). As revealed in 2018, the most frequently detected pathogen was *R. helvetica*, followed by *B. garinii* and *N. mikurensis*. The geographical distribution of the two most common *Borrelia* species, as well as the geographical distribution of *A. phagocytophilum* and *R. helvetica* infections in *I. ricinus* collected in 2019 is presented in Fig. 2, and details of infected ticks identified in each province is provided in Additional file 5.

*Ixodes persulcatus* (nymph, *n* = 1, females, *n* = 189 and males, *n* = 85) showed a 22.9% (18.3–28.2, *n* = 63) infection rate with one up to two different pathogens per tick of the following TBPs, *B. garinii*, *B. afzelii*, *Ehrlichia* spp., *Rickettsia* spp., *R. helvetica* and *B. venatorum*. Infection rate did not differ between female (*n* = 39) and male *I. persulcatus* (*n* = 24; *χ*^*2*^ = 1.9128, *df* = 1, *p* = 0.166). *Borrelia garinii* was the most common pathogen (18.2%, 14.1–23.2, *n* = 50) detected from female and male *I. persulcatus* in 14.3% (10.0–20.0, *n* = 27) and 27.1% (18.8–37.3, *n* = 23), respectively. Results are summarized in detail in Table 2. The geographical distribution of the two most common *Borrelia* species, as well as the geographical distributions of *A. phagocytophilum* and *R. helvetica* infections in *I. persulcatus* collected in 2019 are presented in Fig. 3, and details of infected ticks identified in each province are provided in Additional file 6.

**Table 2:** The occurrence of tick-borne pathogens in Ixodes spp. Ticks collected in 2019

2019 Sample Pathogen	Sample		I. ricinus								I. persulcatus							
			Nymph		Female		Male		Total		Nymph		Female		Male		Total	
	(*n* = 514)	%	(*n* = 2)	%	(*n* = 221)	%	(*n* = 16)	%	(*n* = 239)	%	(*n* = 1)	%	(*n* = 189)	%	(*n* = 85)	%	(*n* = 275)	%
*Borrelia garinii*	59	11.5%			5	2.3%	4	25.0%	9	3.8%			27	14.3%	23	27.1%	50	18.2%
*Borrelia afzelii*	6	1.2%			3	1.4%			3	1.3%			3	1.6%			3	1.1%
*Borrelia miyamotoi*	1	0.2%			1	0.5%			1	0.4%								
*Anaplasma phagocytophillum*	2	0.4%			2	0.9%			2	0.8%								
*Ehrlichia spp.*	1	0.2%											1	0.5%			1	0.4%
*Neoehrlichia mikurensis*	9	1.8%	1	50.0%	8	3.6%			9	3.8%								
*Rickettsia helvetica*	18	3.5%			13	5.9%			13	5.4%			5	2.6%			5	1.8%
Other Rickettsia spp.^*^	8	1.6%					1	6.3%	1	0.4%			7	3.7%			7	2.5%
*Babesia venatorum*	6	1.2%			3	1.4%	2	12.5%	5	2.1%					1	1.2%	1	0.4%
Total infections	110	21.4%	1	50.0%	35	15.8%	7	43.8%	43	18.0%			43	22.8%	24	28.2%	67	24.4%

* Excluding samples positive for *Rickettsia helvetica*

### Co-infections

In 2018, 6.0% (4.8–7.6, *n* = 64) of the 1,059 *I. ricinus* individuals were infected with two pathogens simultaneously. Among 16 different combinations of pathogens co-infections, the most frequently detected co-infection combination was *B. burgdorferi* (s.l.) + *Rickettsia* spp. (*n* = 35) followed by *Rickettsia* spp. + *N. mikurensis* (*n* = 6), *B. burgdorferi* (s.l.) + *Babesia* spp. (*n* = 6), *B. burgdorferi* (s.l.) + *N. mikurensis* (*n* = 6), and *B. burgdorferi* (s.l.) + UUKV (*n* = 5). One *I. persulcatus* showed to be co-infected with *Rickettsia* spp. + *N. mikurensis*.

In 2019, there were two *I. ricinus* co-infected with two pathogens, one co-infected with *B. burgdorferi* (s.l.) and *N. mikurensis*, and the other co-infected with *Rickettsia* spp. and *N. mikurensis*. Four *I. persulcatus* were co-infected with two pathogens, either with *B. burgdorferi* (s.l.) and *Rickettsia* spp. (*n* = 3), or *B. burgdorferi* (s.l.) and *Ehrlichia* spp. (*n* = 1).

### Host association of tick-borne pathogens

In 2018, the overall infection rates detected in *I. ricinus* collected from different hosts were as follows: dogs: 38.3% (34.0–42.8, *n* = 178/465), cats: 43.8% (39.3–48.4, *n* = 201/459), and humans: 52.0% (40.9–62.9, *n* = 39/75; Table 3). The most common TBPs detected in *I. ricinus* collected from dogs were *R. helvetica* (16.6%, 13.5–20.2, *n* = 77) followed by *B. garinii* (4.7%, 3.1–7.1, *n* = 22) and *B. afzelii* (4.5%, 3.0–6.8, *n* = 21). The most common TBPs detected in *I. ricinus* collected from cats were *R. helvetica* (15.7%, 12.6–19.3, *n* = 72) followed by *B. garinii* (7.6%, 5.5–10.4, *n* = 35) and *B. afzelii* (6.1%, 4.3–8.7, *n* = 28). *Ixodes ricinus* collected from humans were most frequently infected with *R. helvetica* (16.0%, 9.4–25.9, *n* = 12) and *B. afzelii* (16.0%, 9.4–25.9, *n* = 12) followed by *B. garinii* (6.7%, 2.9–14.7, *n* = 5). Tick-borne pathogens detected in *I. persulcatus* collected from dogs (*n* = 15) were *B. garinii* (*n* = 1), *R. helvetica* (*n* = 1), and *Rickettsia* spp. (*n* = 1). *Ixodes persulcatus* collected from cats (*n* = 3) were infected with *N. mikurensis* (*n* = 1) or *R. helvetica* (*n* = 2). No *Ixodes persulcatus* ticks were collected from humans in 2018.

**Table 3:** Infection rates of tick-borne pathogens in dogs, cats and humans in 2018

Host	Dog														Cat										Human							
Tick species	*I. ricinus*						*I. persulcatus*						Total ticks		*I. ricinus*						*I. persulcatus*		Total ticks		*I. ricinus*						Total ticks	
	Nymph		Female		Male		Nymph		Female		Male				Nymph		Female		Male		Female				Nymph		Female		Male			
Tick number	(*n* = 2)	%	(*n* = 417)	%	(*n* = 46)	%	(*n* = 1)	%	(*n* = 10)	%	(*n* = 4)	%	(*n* = 480)	%	(*n* = 5)	%	(*n* = 412)	%	(*n* = 42)	%	(*n* = 3)	%	(*n* = 462)	%	(*n* = 27)	%	(*n* = 36)	%	(*n* = 12)	%	(*n* = 75)	%
*Borrelia garinii*			17	4.1%	5	10.9%			1	10.0%			23	4.8%	1	20.0%	27	6.6%	7	16.7%			35	7.6%	1	3.7%	3	8.3%	1	8.3%	5	6.7%
*Borrelia afzelii*			20	4.8%	1	2.2%							21	4.4%	1	20.0%	25	6.1%	2	4.8%			28	6.1%	4	14.8%	6	16.7%	2	16.7%	12	16.0%
*Borrelia valaisiana*			1	0.2%									1	0.2%			8	1.9%	1	2.4%			9	1.9%			1	2.8%	1	8.3%	2	2.7%
*Borrelia miyamotoi*																	3	0.7%					3	0.6%			1	2.8%			1	1.3%
*Anaplasma phagocytophillum*			9	2.2%									9	1.9%			3	0.7%	2	4.8%			5	1.1%					1	8.3%	1	1.3%
Other Anaplasma spp.^*^			1	0.2%									1	0.2%																		
*Neoehrlichia mikurensis*			8	1.9%	1	2.2%							9	1.9%			12	2.9%			1	33.3%	13	2.8%								
*Rickettsia helvetica*			64	15.3%	13	28.3%			1	10.0%			78	16.3%	1	20.0%	65	15.8%	6	14.3%	2	66.7%	74	16.0%	5	18.5%	6	16.7%	1	8.3%	12	16.0%
Other Rickettsia spp.^**^			18	4.3%							1	25.0%	19	4.0%			13	3.2%	4	9.5%			17	3.7%								
*Francisella tularensis*																	1	0.2%					1	0.2%								
*Babesia microti*			1	0.2%									1	0.2%			1	0.2%					1	0.2%								
*Babesia venatorum*			4	1.0%	1	2.2%							5	1.0%			7	1.7%					7	1.5%	2	7.4%	1	2.8%			3	4.0%
*Babesia divergens*			2	0.5%									2	0.4%			1	0.2%					1	0.2%								
*UUKV*			5	1.2%	1	2.2%							6	1.3%			7	1.7%	1	2.4%			8	1.7%			1	2.8%	1	8.3%	2	2.7%

* Excluding samples positive for *Anaplasma phagocytophilum*

** Excluding samples positive for *Rickettsia helvetica*

In 2019, the overall infection rates detected in *I. ricinus* collected from hosts were as follows: dogs: 19.6% (14.1–26.6, *n* = 30/153), cats: 12.3% (6.1–23.2, *n* = 7/57), and humans: 33.3% (9.7–70.0, *n* = 2/6; Table 4). The most common TBPs detected in female *I. ricinus* (*n* = 141) collected from dogs were *R. helvetica* (6.4%, 3.4–11.7, *n* = 9) followed by *B. garinii* (3.5%, 1.5–8.0, *n* = 5) and *N. mikurensis* (3.5%, 1.5–8.0, *n* = 5). The most common TBPs detected in female *I. ricinus* (*n* = 56) collected from cats were *N. mikurensis* (5.4%, 1.8–14.6, *n* = 3) followed by *R. helvetica* (3.6%, 1.0–12.1, *n* = 2). Overall infection rates detected in *I. persulcatus* collected from hosts were as follows: dogs: 22.6% (17.6–28.6, *n* = 50/221), cats 33.3%, (12.1–64.6, *n* = 3/9), and humans 15.8% (5.5–37.6, *n* = 3/19). The majority of *I. persulcatus* was collected from dogs (88.8%, 84.2–92.1, *n* = 221) and the most dominantly detected pathogen found in female (*n* = 146) and male (*n* = 74) *I. persulcatus* collected from dogs was *B. garinii* in 13.0% (8.5–19.4, *n* = 19) and 23.0% (14.9–33.7, *n* = 17), respectively.

**Table 4:** Infection rates of tick-borne pathogens in dogs, cats and humans in 2019

Host	Dog												Cat								Human											
Tick species	*I. ricinus*				*I. persulcatus*						Total ticks		*I. ricinus*				*I. persulcatus*		Total ticks		*I. ricinus*						*I. persulcatus*				Total ticks	
	Female		Male		Nymph		Female		Male				Female		Male		Female				Nymph		Female		Male		Female		Male			
Tick number	(*n* = 141)	%	(*n* = 12)	%	(*n* = 1)	%	(*n* = 146)	%	(*n* = 74)	%	(*n* = 374)	%	(*n* = 56)	%	(*n* = 1)	%	(*n* = 9)	%	(*n* = 66)	%	(*n* = 1)	%	(*n* = 4)	%	(*n* = 1)	%	(*n* = 16)	%	(*n* = 3)	%	(*n* = 25)	%
*Borrelia garinii*	5	3.5%	3	25.0%			19	13.0%	17	23.0%	44	11.8%			1	100.0%	1	11.1%	2	3.0%							2	12.5%			2	8.0%
*Borrelia afzelii*	2	1.4%					2	1.4%			4	1.1%					1	11.1%	1	1.5%			1	25.0%							1	4.0%
*Borrelia miyamotoi*	1	0.7%									1	0.3%																				
*Anaplasma phagocytophillum*	1	0.7%									1	0.3%	1	1.8%					1	1.5%												
*Ehrlichia* spp.							1	0.7%			1	0.3%																				
*Neoehrlichia mikurensis*	5	3.5%									5	1.3%	3	5.4%					3	4.5%												
*Rickettsia helvetica*	9	6.4%					5	3.4%			14	3.7%	2	3.6%					2	3.0%												
Other Rickettsia spp.^*^							5	3.4%			5	1.3%					1	11.1%	1	1.5%					1	100.0%					1	4.0%
*Babesia venatorum*	3	2.1%	1	8.3%							4	1.1%																	1	33.3%	1	4.0%

* Excluding samples positive for *Rickettsia helvetica*

## Discussion

In northern Sweden the increasing abundance of the *I. ricinus* tick population, and the expansion of the *I. persulcatus* population, represent a phenomenon that will change the attitudes of the human communities towards their interaction with the surrounding environment. This geographical area is at the same time the northern distribution limit of *I. ricinus* and the western distribution limit of *I. persulcatus*, and therefore relevant to observe and characterize the diversity of tick-borne pathogens (TBPs) harboured by both these species in a new ecological scenario provided by environmental and climatic changes.

Other studies on *I. ricinus* ticks collected from dogs and cats at similar latitudes were performed in northern Norway; here, *Borrelia* spp. was detected in 13% of specimens (peaking at 29% in the southern part of the study area), *(A) phagocytophilum* was found in 3% of specimens (3.2% in dog-collected and 2.3% in cat-collected specimens) and *N. mikurensis* were detected in 5.6% of the ticks collected (7.0% in dog-collected and 4.1% in cat-collected specimens) [56–58]. The most common species of *Borrelia* detected in a study performed previously [57] was *(B) afzelii*, followed by *B. garinii* and *B. valaisiana*. All the detected pathogens occurred at lower rates in our study, but *I. ricinus* collected in Norway were not infected by *B. spielmanii* and *B. miyamotoi*. Our figures regarding the occurrence of *Borrelia* spp., *B. miyamotoi* and *A. phagocytophilum* are also lower than recently reported data from Finland, where these pathogens were respectively detected in 10.5%, 1.5% and 3.5% of the *I. ricinus* and *I. persulcatus* ticks collected from dogs and cats [59]. In the same study, *B. venatorum* had a similar occurrence (1.2%) and *N. mikurensis* was detected less often (0.9%) [59] compared to our findings. Data from Denmark shows that *I. ricinus* ticks collected from dogs had higher occurrence rates of *Borrelia* spp. (15%, *B. afzelii* being the most common species found) and *Babesia* spp. (8%, *B. microti* being the most common species found), similar occurrence rates of *Rickettsia* spp. (16%, *R. helvetica* being the most commonly found) and lower occurrence of *N. mikurensis* (1%) compared to our results [60].

Regarding human-collected ticks, prevalence of TBEV, *Borrelia* spp., *A. phagocytophilum* and *Rickettsia* spp. was assessed in studies using specimens collected mainly from central and southern Sweden and the Åland islands in Finland [41]. TBEV was found in 0.23% of ticks [42], *Borrelia* spp. was detected in 19–26% of ticks collected from humans, *B. afzelii* being the most frequent species detected (50% of the typeable samples), followed by *B. garinii* (19% of the typeable samples); noteworthy, *B miyamotoi* occurred in 2% of the examined samples [40, 61]. In one of these studies, 11% of ticks collected from northern Sweden were infected by *Borrelia* spp. but the sample size was smaller than those from other regions of Sweden [61]. Regarding other TBPs detected in ticks collected from human patients, *A. phagocytophilum* was present in 1.2% [44], *Babesia* spp. was found in 3.1% [62] and *Rickettsia* spp. was found in 8.3% of the analysed specimens [37]. In our study, except for rickettsiae, that occurred in a higher proportion of analysed samples (14.6%), we observed a lower occurrence of TBPs in *I. ricinus* collected from humans. However, it is important to have in mind that the tick population in the investigated study area is much smaller compared to the populations in southern and central Sweden.

The only available study on the occurrence of TBPs from *I. persulcatus* in northern part of Sweden was performed on questing ticks and showed that *Borrelia* spp. occurred in 55% of the samples, again *B. afzelii* being the most common species identified, followed by *B. garinii* [63]. The only other TBP was *Rickettsia* spp., detected in one *I. persulcatus* tick. It is not surprising that TBPs can occur at different rates in ticks sampled from the field than in the host-feeding ones; while TBEV usually occur at low prevalences in both kinds of specimens [64, 65], the proportion of questing ticks harbouring other TBPs can have a wide range. For example, *Borrelia* spp. was detected in questing *I. ricinus* ticks, between 11% [66] and up to 15–23% in nymphs and adults, with higher occurrences recorded in southern Sweden [67], while rickettsiae are known to occur in 2–22% of questing ticks collected across Sweden [68, 69].

The inclusion of viral TBPs in the microfluidic analysis allowed us to confirm that Uukuniemi virus (UUKV), an apparently non-pathogenic virus, can be found in ticks. The occurrence of this virus was higher in our study than described in a previous study, but in this case the analysed ticks had been collected in southern areas of Sweden [50]. Since *B. garinii* relies mostly on birds as main reservoir hosts, it might be that the relatively high occurrence of this species among other *Borrelia* species recorded in our study is reflecting an important role of birds in the ecology of *I. ricinus* in this rather new distribution area [70]. The sparse occurrence of *F. tularensis* (observed in *I. ricinus* collected in 2018, when the sample size was much larger), confirms that high-throughput techniques can be useful to detect potentially emerging pathogens that could deserve targeted studies in order to gather more information on their circulation and ecology. For example, *F. tularensis* has recently been detected in ticks feeding on hares and cats in Sweden [71].

It is difficult to interpret the role of co-infections, since no clinical data could be recorded from the collectors. It can be speculated that the most commonly occurring co-infection was due to the fact that both *Rickettsia* spp. and *Borrelia* spp. were the most frequently detected TBPs in the present study.

Regarding our study, an inter-year variation of the results cannot be provided, since the two study areas and consequently the tick species collected differ between 2018 and 2019, *I. persulcatus* representing around half of the specimens collected in 2019, while this species represented only 0.8% of the collected specimens in 2018 [6]. The difference of collected species is also reflected in the reported occurrence of different TBPs, i.e. the most frequently detected pathogen in 2018 was *R. helvetica* (detected in *I. ricinus* ticks), while in 2019 it was *B. garinii* (detected in *I. persulcatus* ticks). While analysing questing ticks can provide more accurate figures on the occurrence of TBPs in ticks, flagging would not have been feasible for such large areas as the ones covered by the present study. High-throughput PCR analyses such as those used in the present study instead provided a rare opportunity to detect a wide array of pathogens in a vast region previously largely lacking information on the distribution of TBPs. These studies are in line with a One Health approach where sample collection, analyses and interpretation of the results are based on both animal and human sources.

## Conclusions

This study provides data on TBPs from a rather poorly investigated geographical area using a One Health perspective, since ticks collected from both animal and human hosts were included in the study materials. As the host-feeding tick can harbor TBPs’ nucleic acids acquired directly during the feeding activity, the presented figures could over-represent the occurrence of some TBPs, and underrepresent the occurrence of others e.g. *Borrelia* spp., that occurs at lower levels in engorged ticks collected from pets, so they must be interpreted with caution. At the same time, microfluidic techniques are confirmed to be an effective tool to screen large amounts of samples and to find a large array of TBPs, even ones occurring at lower rates. The combination of a citizen science approach allowing sampling across large areas and wide molecular screening techniques represents an effective way to design updated risk maps. Furthermore, it allows finding potential TBP foci that deserve targeted sampling in order to obtain a more accurate risk assessment and disease prevention both for the humans and animals inhabiting the large study area.

## Supplementary Information

Supplementary Material 1. Table S1. List of primers/probe sets, target genes, amplicon size and references for the PCRs used in the high-throughput microfluidic real-time PCR.

Supplementary Material 2. Table S2. Primers used for validation of confirmation of microfluidic real-time PCR results.

Supplementary Material 3. Table S3. Consensus sequences were analyzed using Basic Local Alignment Search Tool (BLAST) at the website of the National Center for Biotechnology Information (NCBI) (https://ncbi.nlm.nih.gov/blast) and compared to reference sequences listed in the GenBank sequence database (NCBI). Accession numbers for the submitted sequences are reported here.

Supplementary Material 4. Table S4. Details of infected *Ixodes ricinus* identified in each province 2018. Table S5. Details of infected *Ixodes ricinus* identified in each province 2019. Table S6. Details of infected *Ixodes persulcatus* identified in each province 2019. 

## Data Availability

All datasets generated and analysed during the study are included in the manuscript. The generated and confirmed sequences from the present study have been submitted to GenBank, accession numbers being provided in Additional File 3.
