# Supplementary material for: High-throughput screening for tick-borne pathogens in Ixodid ticks collected through crowdsourcing in northern Sweden
Source: Acta Vet Scand. 2026 Feb 18;68:14. doi: 10.1186/s13028-026-00854-9 (PMC12931012; doi:10.1186/s13028-026-00854-9)
Supplement: Supplementary file 4 — Supplementary Material 4. Table S4. Details of infected Ixodes ricinus identified in each province 2018. Table S5. Details of infected Ixodes ricinus identified in each province 2019. Table S6. Details of infected Ixodes persulcatus identified in each province 2019. [file 13028_2026_854_MOESM4_ESM.docx]

| Table S4. Infection of tick-borne pathogens in *Ixodes ricinus* collected from northern Sweden in 2018. | | | | | | | | | | | | | | | | | | | | |  | | | | |  |  |
| --- | --- | --- | --- | --- | --- | --- | --- | --- | --- | --- | --- | --- | --- | --- | --- | --- | --- | --- | --- | --- | --- | --- | --- | --- | --- | --- | --- |
| Province | Stage | No. of ticks examined | No. of positive ticks (%) | | *Borrelia* | | | | | | | | *Anaplasma* | | | | | | *Ehrlichia* | |  | |  | |  |  |  |
|  |  |  |  |  | *Borrelia garinii* | *Borrelia afzelii* | | *Borrelia valaisiana* | | | *Borrelia miyamotoi* | | *Anaplasma phagocytophilum* | | | | *Anaplasma spp.* | | *Candidatus Neoehrlichia mikurensis* | |  |  | |  |  |  |  |
| Lappland | FF^a^ | 18 | 11 (61.1) | |  | 1 (5.6) | |  | | |  | | | |  | |  | | 2 (11.1) | |  |  | |  |  |  |  |
|  | MM^b^ | 1 |  | |  |  | |  | | |  | | | |  | |  | |  | |  |  | |  |  |  |  |
| Norrbotten | FF | 37 | 9 (24.3) | |  | 2 (5.4) | |  | | |  | | | |  | |  | | 1 (2.7) | |  |  | |  |  |  |  |
| Västerbotten | FF | 78 | 26 (33.3) | | 4 (5,1) | 3 (3.8) | | 1 (1.3) | | |  | | | | 2 (2.6) | |  | | 1 (1.3) | |  |  | |  |  |  |  |
|  | MM | 4 | 2 (50.0) | |  |  | |  | | |  | | | |  | |  | |  | |  |  | |  |  |  |  |
| Jämtland | NN^c^ | 1 | 1 (100.0) | |  | 1 (100.0) | |  | | |  | | | |  | |  | |  | |  |  | |  |  |  |  |
|  | FF | 38 | 13 (34.2) | | 1 (2.6) | 1 (2.6) | | 1 (2.6) | | |  | | | | 1 (2.6) | |  | | 2 (5.3) | |  |  | |  |  |  |  |
|  | MM | 1 |  | |  |  | |  | | |  | | | |  | |  | |  | |  |  | |  |  |  |  |
| Ångermanland | NN | 3 | 1 (33.3) | |  |  | |  | | |  | | | |  | |  | |  | |  |  | |  |  |  |  |
|  | FF | 165 | 43 (26.1) | | 5 (3.0) | 2 (1.2) | | 4 (2.4) | | | 1 (0.6) | | | | 3 (1.8) | | 1 (0.6) | | 2 (1.2) | |  |  | |  |  |  |  |
|  | MM | 12 | 3 (25.0) | | 1 (8.3) |  | | 1 (8.3) | | |  | | | |  | |  | |  | |  |  | |  |  |  |  |
| Härjedalen | FF | 10 | 3 (30.0) | |  | 1 (10.0) | |  | | |  | | | |  | |  | | 1 (10.0) | |  |  | |  |  |  |  |
| Medelpad | NN | 4 | 1 (25.0) | | 1 (25.0) |  | |  | | |  | | | |  | |  | |  | |  |  | |  |  |  |  |
|  | FF | 125 | 45 (36.0) | | 12 (9.6) | 11 (8.8) | | 1 (0.8) | | |  | | | | 3 (2.4) | |  | | 2 (1.6) | |  |  | |  |  |  |  |
|  | MM | 19 | 9 (47.4) | | 4 (21.1) | 1 (5.3) | | 1 (5.3) | | |  | | | |  | |  | |  | |  |  | |  |  |  |  |
| Hälsingland | NN | 14 | 5 (35.7) | |  | 2 (14.3) | |  | | |  | | | |  | |  | |  | |  |  | |  |  |  |  |
|  | FF | 238 | 77 (32.4) | | 14 (5.9) | 13 (5.5) | | 3 (1.3) | | | 1 (0.4) | | | | 1 (0.4) | |  | | 4 (1.7) | |  |  | |  |  |  |  |
|  | MM | 35 | 14 (40.0) | | 2 (5.7) | 1 (2.9) | | 1 (2.9) | | |  | | | | 2 (5.7) | |  | |  | |  |  | |  |  |  |  |
| Dalarna | NN | 2 | 1 (50.0) | |  |  | |  | | |  | | | |  | |  | |  | |  |  | |  |  |  |  |
|  | FF | 112 | 37 (33.0) | | 3 (2.7) | 5 (4.5) | | 1 (0.9) | | | 1 (0.9) | | | | 4 (3.6) | |  | | 4 (3.6) | |  |  | |  |  |  |  |
|  | MM | 15 | 4 (26.7) | |  | 1 (6.7) | |  | | |  | | | |  | |  | |  | |  |  | |  |  |  |  |
| Gästrikland | NN | 10 | 4 (40.0) | | 1 (10.0) | 2 (20.0) | |  | | |  | | | |  | |  | |  | |  |  | |  |  |  |  |
|  | FF | 93 | 38 (40.9) | | 9 (9.7) | 12 (12.9) | | 1 (1.1) | | | 1 (1.1) | | | | 2 (2.2) | |  | | 5 (5.4) | |  |  | |  |  |  |  |
|  | MM | 19 | 13 (68.4) | | 6 (31.6) | 2 (10.5) | |  | | |  | | | | 1 (5.3) | |  | | 1 (5.3) | |  |  | |  |  |  |  |
| Uppland | FF | 4 | 1 (25.0) | |  | 1 (25.0) | |  | | |  | | | |  | |  | |  | |  |  | |  |  |  |  |
|  | MM | 1 |  | |  |  | |  | | |  | | | |  | |  | |  | |  |  | |  |  |  |  |
| Total |  | 1059 | 361 (34.1) | | 63 (5.9) | 62 (5.9) | | 15 (1.4) | | | 4 (0.4) | | | | 19 (1.8) | | 1 (0.1) | | 25 (2.4) | |  |  | |  |  |  |  |
|  | | | | | | | | | | | | | | | | | | | | |  | | | | |  |  |
|  |  | | | | | | | | | | | | | | | | | | | |  | | | | |  |  |
| Continue | | | |  | | | | | |  | | | | | | | | | |  |  | | | | |  |  |
| Province | Stage | No. of ticks examined | No. of positive ticks (%) | | *Rickettsia* | | | | *Francisella* | | | *Babesia* | | | | | | *Uukuvirus* | |  | | | | | |  |  |
|  |  |  |  |  | *Rickettsia helvetica* | | *Rickettsia spp.* | | *Francisella tularensis* | | | *Babesia microti* | | *Babesia venatorum* | | *Babesia divergens* | | *Uukuniemi virus* | |  | | | | | |  |  |
| Lappland | FF | 18 | 11 (61.1) | | 8 (44.4) | |  | |  | | |  | |  | |  | | 1 (5.6) | |  | | | | | |  |  |
|  | MM | 1 |  | |  | |  | |  | | |  | |  | |  | |  | |  | | | | | |  |  |
| Norrbotten | FF | 37 | 9 (24.3) | | 4 (10.8) | | 2 (5.4) | |  | | |  | |  | | 1 (2.7) | |  | |  | | | | | |  |  |
| Västerbotten | FF | 78 | 26 (33.3) | | 12 (15.4) | | 2 (2.6) | |  | | |  | | 1 (1.3) | |  | | 2 (2.6) | |  | | | | | |  |  |
|  | MM | 4 | 2 (50.0) | | 1 (25.0) | |  | |  | | |  | |  | |  | | 1 (25.0) | |  | | | | | |  |  |
| Jämtland | NN | 1 | 1 (100.0) | |  | |  | |  | | |  | |  | |  | |  | |  | | | | | |  |  |
|  | FF | 38 | 13 (34.2) | | 7 (18.4) | | 1 (2.6) | |  | | |  | |  | |  | | 1 (2.6) | |  | | | | | |  |  |
|  | MM | 1 |  | |  | |  | |  | | |  | |  | |  | |  | |  | | | | | |  |  |
| Ångermanland | NN | 3 | 1 (33.3) | | 1 (33.3) | |  | |  | | |  | |  | |  | |  | |  | | | | | |  |  |
|  | FF | 165 | 43 (26.1) | | 22 (13.3) | | 5 (3.0) | |  | | |  | | 2 (1.2) | | 1 (0.6) | |  | |  | | | | | |  |  |
|  | MM | 12 | 3 (25.0) | |  | | 1 (8.3) | |  | | |  | |  | |  | |  | |  | | | | | |  |  |
| Härjedalen | FF | 10 | 3 (30.0) | | 2 (20.0) | |  | |  | | |  | |  | |  | |  | |  | | | | | |  |  |
| Medelpad | NN | 4 | 1 (25.0) | |  | |  | |  | | |  | |  | |  | |  | |  | | | | | |  |  |
|  | FF | 125 | 45 (36.0) | | 15 (12.0) | | 7 (5.6) | |  | | |  | | 3 (2.4) | |  | |  | |  | | | | | |  |  |
|  | MM | 19 | 9 (47.4) | | 2 (10.5) | | 1 (5.3) | |  | | |  | |  | |  | |  | |  | | | | | |  |  |
| Hälsingland | NN | 14 | 5 (35.7) | | 2(14.3) | |  | |  | | |  | | 1 (7.1) | |  | |  | |  | | | | | |  |  |
|  | FF | 238 | 77 (32.4) | | 37 (15.5) | | 8 (3.4) | |  | | |  | | 6 (2.5) | | 1 (0.4) | | 2 (0.8) | |  | | | | | |  |  |
|  | MM | 35 | 14 (40.0) | | 7 (20.0) | | 1 (2.9) | |  | | |  | |  | |  | |  | |  | | | | | |  |  |
| Dalarna | NN | 2 | 1 (50.0) | | 1 (50.0) | |  | |  | | |  | |  | |  | |  | |  | | | | | |  |  |
|  | FF | 112 | 37 (33.0) | | 15 (13.4) | | 4 (3.6) | | 1 (0.9) | | | 2 (1.8) | | 3 (2.7) | |  | |  | |  | | | | | |  |  |
|  | MM | 15 | 4 (26.7) | | 3 (20.0) | |  | |  | | |  | | 1 (6.7) | |  | |  | |  | | | | | |  |  |
| Gästrikland | NN | 10 | 4 (40.0) | | 2 (20.0) | |  | |  | | |  | | 1 (10.0) | |  | |  | |  | | | | | |  |  |
|  | FF | 93 | 38 (40.9) | | 19 (20.4) | | 2 (2.2) | |  | | | 1 (1.1) | |  | |  | | 7 (7.5) | |  | | | | | |  |  |
|  | MM | 19 | 13 (68.4) | | 7 (36.8) | | 1 (5.3) | |  | | |  | |  | |  | | 2 (10.5) | |  | | | | | |  |  |
| Uppland | FF | 4 | 1 (25.0) | |  | |  | |  | | | 1 (25.0) | |  | |  | |  | |  | | | | | |  |  |
|  | MM | 1 |  | |  | |  | |  | | |  | |  | |  | |  | |  | | | | | |  |  |
| Total |  | 1059 | 361 (34.1) | | 167 (15.8) | | 35 (3.3) | | 1 (0.1) | | | 4 (0.4) | | 18 (1.7) | | 3 (0.3) | | 16 (1.5) | |  | | | | | |  |  |
| a Female, b Male, c Nymph | | | | | | | | | | | | | | | | | | | |  | | | | | |  |  |

| Table S5. Infection of tick-borne pathogens in *Ixodes ricinus* collected from northern Sweden in 2019 | | | | | | | | | | | |
| --- | --- | --- | --- | --- | --- | --- | --- | --- | --- | --- | --- |
| Location | Stage | No. of ticks examined | No. of positive ticks (%) | *Borrelia* | | | *Anaplasma* | *Ehrlichia* | *Rickettsia* | | *Babesia* |
|  |  |  |  | *Borrelia garinii* | *Borrelia afzelii* | *Borrelia miyamotoi* | *Anaplasma phagocytophilum* | *Candidatus Neoehrlichia mikurensis* | *Rickettsia helvetica* | *Rickettsia spp.* | *Babesia venatorum* |
| Lappland | FF^a^ | 9 | 2 (22.2) | 1 (11.1) |  |  |  |  | 1 (11.1) |  |  |
| Norrbotten | FF | 52 | 8 (15.4) | 3 (5.8) | 2 (25.0) | 1 (1.9) | 1 (1.9) |  | 1 (1.9) |  |  |
|  | MM^b^ | 4 |  |  |  |  |  |  |  |  |  |
| Västerbotten | NN^c^ | 2 | 1 (50.0) |  |  |  |  | 1 (50.0) |  |  |  |
|  | FF | 101 | 15 (14.9) | 1 (1.0) |  |  | 1 (1.0) | 2 (2.0) | 8 (7.9) |  | 3 (3.0) |
|  | MM | 6 | 3 (50.0) | 1 (16.7) |  |  |  |  |  |  | 2 (33.3) |
| Jämtland | FF | 59 | 8 (13.6) |  | 1 (1.7) |  |  | 6 (10.2) | 3 (5.1) |  |  |
|  | MM | 6 | 4 (66.7) | 3 (50.0) |  |  |  |  |  | 1 (16.7) |  |
| Total |  | 239 | 41 (17.2) | 9 (3.8) | 3 (1.3) | 1 (0.4) | 2 (0.8) | 9 (3.8) | 13 (5.4) | 1 (0.4) | 5 (2.1) |
| a Female, b Male, c Nymph | | | | | | | | | | | |

| Table S6. Infection of tick-borne pathogens in *Ixodes persulcatus* collected from northern Sweden in 2019 | | | | | | | | | | | | |  |
| --- | --- | --- | --- | --- | --- | --- | --- | --- | --- | --- | --- | --- | --- |
| Location | Stage | No. of ticks examined | No. of positive ticks (%) | *Borrelia* | | | *Ehrlichia* | | *Rickettsia* | | | *Babesia* | |
|  |  |  |  | *Borrelia garinii* | *Borrelia afzelii* | *Ehrlichia spp.* | | *Rickettsia helvetica* | | *Rickettsia spp.* | *Babesia venatorum* | |  |
| Lappland | FF^a^ | 10 |  |  |  |  | |  | |  |  | |  |
| Norrbotten | NN^c^ | 1 |  |  |  |  | |  | |  |  | |  |
|  | FF | 172 | 40 (23.3) | 27 (15.7) | 3 (1.7) | 1 (0.6) | | 5 (2.9) | | 7 (4.1) |  | |  |
|  | MM^b^ | 84 | 23 (27.4) | 23 (27.4) |  |  | |  | |  |  | |  |
| Västerbotten | FF | 5 |  |  |  |  | |  | |  |  | |  |
|  | MM | 1 | 1 (100.0) |  |  |  | |  | |  | 1 (100.0) | |  |
| Jämtland | FF | 2 |  |  |  |  | |  | |  |  | |  |
| Total |  | 275 | 64 (23.3) | 50 (18.2) | 3 (1.1) | 1 (0.4) | | 5 (1.8) | | 7 (2.5) | 1 (0.4) | |  |
| a Female, b Male, c Nymph | | | | | | | | | | | | |  |
